# Association between control group therapy and magnitude of clinical benefit of cancer drugs

**DOI:** 10.1038/s41598-022-25983-9

**Published:** 2022-12-09

**Authors:** Consolacion Molto, Ariadna Tibau, Aida Bujosa, Jose Carlos Tapia, Abhenil Mittal, Faris Tamimi, Eitan Amir

**Affiliations:** 1 Division of Medical Oncology and Hematology, Department of Medicine, Princess Margaret Cancer Centre and University of Toronto, 610 University Ave, Toronto, ON M5G 2M9 Canada; 2 Medical Oncology Department, Hospital de la Santa Creu i Sant Pau, Department of Medicine of Universitat Autònoma de Barcelona, Institut d’investigacio Biomedica Sant Pau, Barcelona, Catalonia Spain; 3 Medical Oncology Department, Hospital Son Llàtzer, Palma de Mallorca, Balearic Islands Spain

**Keywords:** Oncology, Public health

## Abstract

Little is known about the impact of control group therapy on clinical benefit scales such as American Society of Clinical Oncology Value Framework (ASCO-VF), European Society for Medical Oncology Magnitude Clinical Benefit Scale (ESMO-MCBS), National Comprehensive Cancer Network (NCCN) Evidence Blocks and ASCO Cancer Research Committee (ASCO-CRC). We searched Drugs@FDA to identify cancer drugs approved between January 2012 and December 2021 based on randomized trials (RCTs). Definition of substantial clinical benefit was based on recommendations for each scale. Associations between characteristics of control group therapy and clinical benefit were explored using logistic regression. RCTs with a control group of active treatment plus placebo were associated with significantly lower odds of substantial benefit with ESMO-MCBS (OR 0.27, P = 0.003) and ASCO-VF (OR 0.30, P = 0.008) but not with NCCN Evidence Blocks or ASCO-CRC. This effect was attenuated and lost statistical significance without adjustment for quality of life (QoL) and/or toxicity (ESMO-MCBS OR 0.50, P = 0.17; ASCO-VF OR 0.49, P = 0.11). Clinical benefit scales can be sensitive to control group therapy. RCTs with substantial overlap between experimental and control therapy showed lower magnitude of clinical benefit using ESMO-MCBS and ASCO-VF scales; possibly due to differences in the weighting of QoL and toxicity between different frameworks.

## Introduction

Several oncology societies have developed tools to quantify the magnitude of clinical benefit of drugs for the treatment of solid tumors. These include the American Society of Clinical Oncology Value Framework (ASCO-VF)^1,2^, the European Society for Medical Oncology Magnitude of Clinical Benefit Scale (ESMO-MCBS)^3,4^, the National Comprehensive Cancer Network (NCCN) Evidence Blocks^5^ and the ASCO Cancer Research Committee criteria (ASCO-CRC)^6^.

The magnitude of clinical benefit does not influence regulatory approval of cancer drugs. Approval by regulatory agencies such as the US Food and Drug Administration (FDA) requires substantial evidence of safety and efficacy from adequate and well-controlled trials irrespective of the magnitude of such benefit^7^. Advances in the understanding of the molecular basis of cancer, has led to rapid development of new drugs and an increasing number of cancer drug approvals based on non-randomized trials^8,9^. Despite this, randomized controlled trials (RCTs) remain the gold standard to evaluate the benefits and risks of new cancer therapies^10^. However, RCTs have limitations including the increasing use of intermediate endpoints which have not been validated as true surrogates for definitive outcomes^11,12^ and the choice of control group therapy which may not always reflect contemporary standard of care^13–16^.

While there is an extensive literature exploring the associations between the magnitude of clinical benefit and characteristics of new drugs as well as the clinical trial design supporting their approval^9,17–19^ much less is known about the influence of control group therapy on the output of clinical benefit frameworks. Knowledge of the impact of control group therapy could aid in the design of clinical trials (such as expected effect size and influence^17^ on quality of life (QoL) assessment), inform drug reimbursement decisions by payers and provide feedback to the developers of value frameworks. In this article, we quantify the proportion of RCTs meeting thresholds for substantial clinical benefit at the time of FDA marketing approval and assess the association between characteristics of control group therapy and magnitude of clinical benefit. We hypothesized that the magnitude of clinical benefit difference will be greater in trials in which control group therapy has minimal or no overlap with the experimental group.

## Methods

### Data sources

We searched the Drugs@FDA database^20,21^ to identify applications for approvals of cancer drugs for solid tumors from January 1, 2012, to December 31, 2021. We excluded drugs approved for hematologic malignancies, for pediatric populations and non-therapeutic agents such as medical devices and diagnostic or contrast agents. Then, we excluded applications which were based exclusively on single-arm or non-randomized trials as well as non-inferiority or equivalence studies. Finally, we searched MEDLINE (host: PubMed)^22^ to identify primary publications of clinical trials supporting FDA approvals.

### Data extraction

Four authors (C.M., J.C.T., A.B. and A.T.) extracted data using predesigned electronic forms. The following characteristics were collected for each application: approval date, approval indication, drug and brand name, cancer site, number of trials supporting approval (one vs. more than one trial), application number, whether or not approval was based on a subgroup analysis, submission type (initial vs. supplemental), type of approval (accelerated vs. regular approval) and regulatory pathways, including priority or standard review, breakthrough or non-breakthrough therapy designation, and orphan or non-orphan drug designation, as determined by the FDA^20,21,23,24^. We paid specific attention to drug class (chemotherapy, hormone therapy, immunotherapy, or targeted therapy) for both the experimental and control groups, defined by the authors. Control arm therapy was divided in two different groups: (1) active treatment group defined as control arm comprising an active anticancer drug such as chemotherapy, hormone therapy, immunotherapy and/or targeted therapy and (2) non-active treatment group defined as control arm comprising placebo and/or best supportive care alone. Subsequently, the active treatment group was further divided into (1) active treatment plus placebo and (2) active treatment without placebo. Among the active treatment plus placebo subgroup, matched placebo was defined as a RCT in which there was overlap between experimental and control arm active treatments with the control arm containing a placebo while the experimental arm comprised an additional active experimental therapy (e.g. chemotherapy plus immunotherapy in the experimental arm vs. the same chemotherapy regimen plus placebo in the control arm)^25^. Finally, when possible, we assessed the quality of the control group therapy (optimal vs. suboptimal) and considered suboptimal control group therapy if prior RCT data showed that the control agent was inferior to an available alternative based on methods reported previously^13^.

We also collected data on whether a companion diagnostic test was available, as defined by the FDA^26^. In addition, the following characteristics were collected for each RCT: setting (curative vs. palliative), study design (open-label vs. blinded), phase (II vs. III), sample size, crossover, and efficacy primary endpoint (Overall survival [OS] vs. intermediate endpoint as defined by the FDA^27^). For RCTs with co-primary endpoints, we identified the most definitive primary endpoint chosen by FDA to support approval. Finally, toxicity data were extracted from published articles and when available so were QoL data. A drug was considered to have shown a QoL benefit if a statistically significant difference was reported between the experimental arm and baseline among RCTs based on a global score, a subscale, or a specific item from a validated patient-reported outcome instrument.

### Data scoring

Three authors (C.M., J.C.T. and A.B.) scored each RCT with 4 different frameworks: ESMO-MCBS version 1.1^4^, ASCO-VF version 2^2^, NCCN Evidence Blocks^5^, and ASCO-CRC^6^. Discrepancies were resolved by a fourth author (A.T.). If more than one RCT supported a single application, each trial was evaluated separately and assigned a separate grade.

Substantial clinical benefit was defined as recommended in prior studies. The ASCO-CRC published targets for clinically meaningful benefit using a single cutoff in clinical trials for 4 cancer types (pancreatic cancer, lung cancer, triple-negative breast cancer, and colon cancer): OS improvements ranging from 2.5 to 6 months and progression-free survival (PFS) improvements ranging from 3 to 5 months. Consistent with prior studies^18,28^, we expanded this definition to RCTs of all solid tumors in the palliative setting^28^. For other scales, the following cutoffs were utilized: ASCO-VF threshold score ≥ 45 (applied in palliative and curative setting)^29^; NCCN Evidence Blocks threshold score ≥ 16 (applied in palliative and curative setting)^18^; and a grade of A or B for trials of curative intent and 4 or 5 for those of non-curative intent using ESMO-MCBS^3,4^.

### Statistical analysis

Data were reported descriptively as proportions, medians, and ranges. Associations between characteristics of control group therapy and substantial clinical benefit scores were explored using logistic regression as were associations between application and clinical trial characteristics and magnitude of clinical benefit. Multivariable analysis was planned only if there were sufficient data to fit a multivariable model adequately. Results of logistic regression were reported as odds ratios (ORs) and their respective 95% confidence intervals (CIs). Sensitivity analyses were performed excluding trials in the curative setting. Additionally, a post-hoc sensitivity analysis was performed to examine the role of QoL and toxicity on substantial benefit measured by ESMO-MCBS and ASCO-VF. In this analysis, we rescored trials without QoL and toxicity data and repeated the analyses described above. All analyses were conducted using SPSS Statistics, version 25 (IBM Corp, Armonk NY). Statistical tests were 2-sided, and statistical significance was defined as a 2-tailed *P* value < 0.05.

### Use of experimental animals and/or human participants’ statement

Live animals and/or humans were not involved in this study.

## Results

### Study cohort

**Figure 1:**
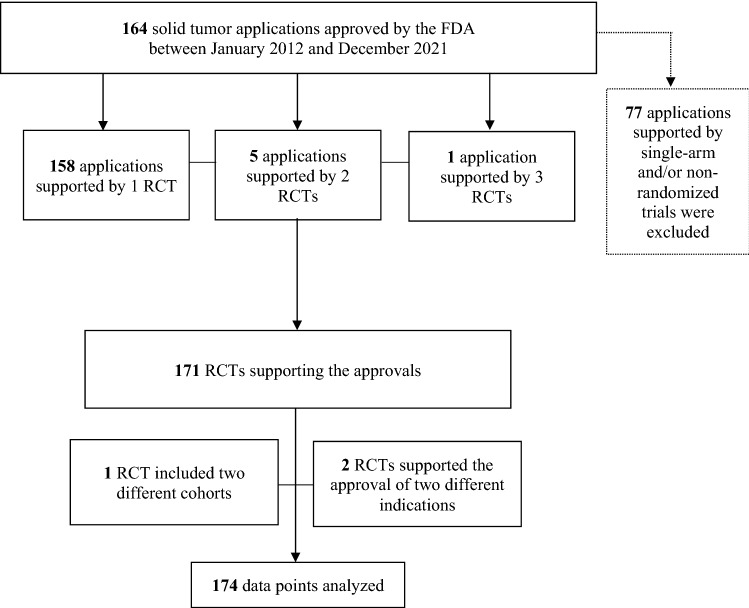
Summary of applications (n = 164), RCTs (n = 171) supporting the FDA application approvals and final data points (n = 174) analyzed in our study. *RCTs* Randomized Controlled Trials, *FDA* US Food and Drug Administration.

We identified 171 RCTs supporting the approval of 76 new cancer drugs for 164 solid tumor indications between January 1, 2012, and December 31, 2021. Among the 164 applications, in 158 (96%) the approval was based on 1 RCT, in 5 (3%) applications the approval was based on 2 RCTs and in 1 application (1%) the approval was based on 3 RCTs. Of the 171 RCTs included, one trial included two different cohorts (germline, and non-germline BRCA mutation carriers)^30^ and two trials each supported approval of two different indications (one trial for pembrolizumab plus chemotherapy and pembrolizumab as a single agent^31^ and another trial for nivolumab plus ipilimumab and nivolumab as a single agent^32^). Consequently, a total of 174 data points were available for analysis (see Fig. 1).

Tables 1 and 2 summarize the characteristics of included applications and trials supporting drug approval at the time of market authorization.

**Table 1:** Characteristics of applications.

Characteristics		*N* (%)
		164 (100)
Submission type	Initial	44 (27)
	Supplemental	120 (73)
Type of approval	Regular	154 (94)
	Accelerated	10 (6)
Breakthrough therapy designation	Yes	46/153 (30)
	No	107/153 (70)
Priority review	Yes	127/158 (80)
	No	31/158 (20)
Orphan drug designation	Yes	74/162 (46)
	No	88/162 (54)
Companion diagnostic	Yes	51 (31)
	No	113 (69)
Number of trials supporting approval	One	158 (96)
	More than one	6 (4)
Approval based on subgroup analysis	Yes	33 (20)
	No	131 (80)
Drug class	Chemotherapy and hormone therapy	17 (10)
	Immunotherapy and targeted therapy	145 (89)
	Other	2 (1)
Primary cancer site	Lung, breast, colorectal, prostate	81 (49)
	Other	83 (51)

**Table 2:** Characteristics of RCTs.

Characteristics		*N* (%)
		174 (100)
Sample size, median (range)		696 (117–5637)
Setting	Palliative	155 (89)
	Curative	19 (11)
Trial design	Open-label	89 (51)
	Double-blind	85 (49)
Phase	III	161 (93)
	II	13 (7)
Crossover	Yes	49/143 (34)
	No	94/143 (66)
Intermediate endpoint	Yes	109 (63)
	No	65 (37)
QoL	Benefit	23/69 (33)
	No benefit	46/69 (64)

*RCTs* Randomized Controlled Trials, *QoL* Quality of Life.

### Value framework scores

The ESMO-MCBS version 1.1 scores could be applied to 172 of 174 trials (99%). Among these, 77 trials (45%) met the threshold for substantial clinical benefit. ASCO-VF version 2 scores were applied to 170 of 174 trials (98%). Of these, 79 trials (46%) met the ASCO-VF scores for substantial clinical benefit. NCCN Evidence Blocks were applied to 150 trials (86%) of which 108 (72%) met the threshold for high clinical benefit. Finally, ASCO-CRC criteria were applicable to 135 (76%) trials in the noncurative setting. Of these, 99 (73%) met the criteria for substantial clinical benefit. When we rescored trials without QoL and toxicity data using ESMO-MCBS and ASCO-VF, 49 (28%) and 68 (40%) trials met the threshold for substantial clinical benefit, respectively.

### Association between control group therapy and clinical benefit

**Figure 2:**
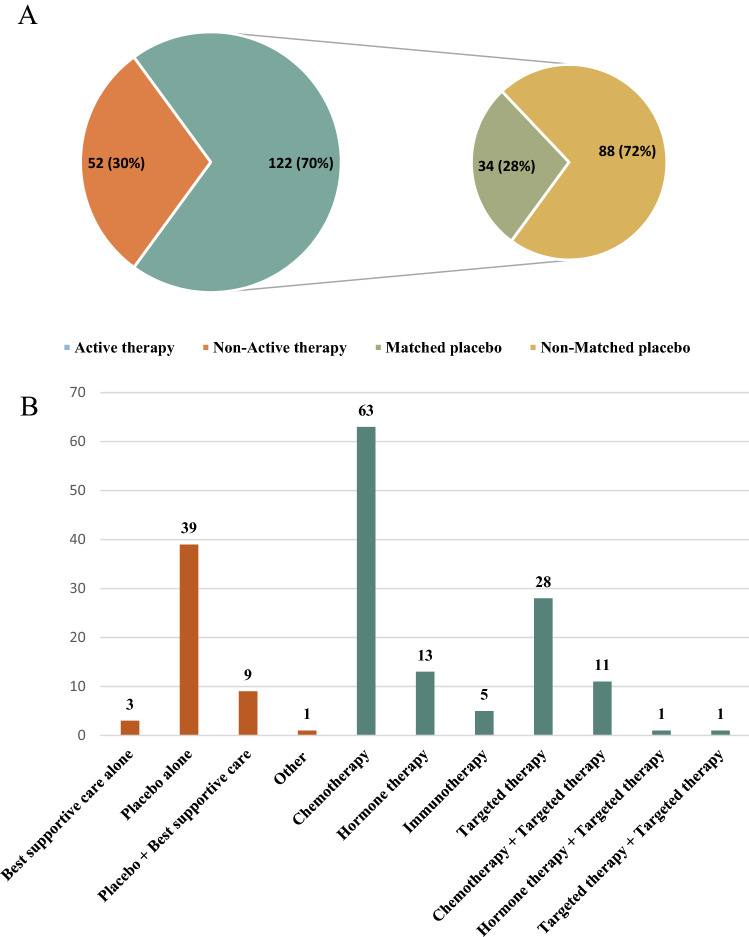
Types of control group therapy. (**A**) Of 174 RCTs analyzed, 70% had an active therapy and 30% had non-active therapy in the control arm. Among RCTs with active therapy in the control arm, 28% were matched placebo (e.g., active therapy plus placebo in the control arm vs. the same active therapy plus an additional drug in the experimental arm). (**B**) Active therapy group was defined as control arm comprising an active anticancer drug such as chemotherapy, hormone therapy, immunotherapy and/or targeted therapy and non-active therapy group was defined as control arm comprising placebo and/or best supportive care alone. Other = Granulocyte–macrophage colony-stimulating factor. *RCTs* Randomized Controlled Trials.

Of the 174 RCTs included in the analysis, 52 (30%) had non-active treatment such as placebo and/or BSC in the control arm and 122 (70%) had an active treatment within the control arm such as chemotherapy, hormone therapy, immunotherapy and/or targeted therapy. Among RCTs with active treatment plus placebo in the control arm, 34 (28%) were matched placebo (see Fig. 2). In total, 17 (10%) trial used a suboptimal control group therapy.

Table 3 shows associations between characteristics of control group therapy and clinical benefit. In univariable analyses, there were non-significant associations between active therapy and higher clinical benefit scores with ESMO-MCBS and NCCN Evidence Blocks, but not with ASCO-VF and ASCO-CRC. RCTs with substantial overlap between experimental and control arms (e.g. a control arm comprising of active treatment plus a matched placebo compared to the same therapy with an additional drug in the experimental arm) were associated with significantly lower odds of substantial benefit with ESMO-MCBS and ASCO-VF (OR 0.27, 95% CI 0.11–0.65; *P* = 0.003 and OR 0.30, 95% CI 0.13–0.73; *P* = 0.008, respectively) but not with NCCN Evidence Blocks or ASCO-CRC criteria (OR 0.74, 95% CI 0.28–1.97; *P* = 0.55 and OR 1.36, 95% CI 0.51–3.64; *P* = 0.54, respectively). Similar results were observed when excluding trials in the curative setting. In the post-hoc sensitivity analysis in which we rescored trials with the ESMO-MCBS and ASCO-VF scales without QoL and/or toxicity adjustment, the magnitude of effect was attenuated, and statistical significance was lost (OR 0.50, 95% CI 0.18–1.34; *P* = 0.17 and OR 0.49, 95% CI 0.20–1.17; *P* = 0.11, respectively). There was no significant difference between type of active therapy and clinical benefit, although, there was a non-significant association with higher odds of substantial benefit with ESMO-MCBS with trials in which the control group therapy was chemotherapy, while for ASCO-CRC a non-significant association in the opposite direction was observed. Analysis of optimal versus suboptimal control group therapy was limited by small number of RCT categorized as having suboptimal control groups. There appeared to be non-significant association with lower magnitude of clinical benefit for trials with optimal control groups using ESMO-MCBS and ASCO-VF. A non-significant effect in the opposite direction was observed for NCCN Evidence Blocks. No association was observed for ASCO-CRC. Multivariable analysis was attempted, but a model could not be fitted adequately.

**Table 3:** Association between characteristics of control group therapy and clinical benefit.

	ESMO-MCBS		ASCO-VF		NCCN evidence blocks		ASCO-CRC	
	OR (95% CI)	*P* Value	OR (95% CI)	*P* Value	OR (95% CI)	*P* Value	OR (95% CI)	*P* Value
**Univariable analysis**^a^								
Active therapy (vs. non-active therapy)	1.63 (0.83–3.17)	0.16	1.14 (0.59–2.19)	0.70	2.04 (0.96–4.34)	0.06	0.80 (0.31–2.06)	0.64
Same active therapy with a matched placebo (vs. different active therapy without placebo)	**0.27** **(0.11**–**0.65)**	**0.003**	**0.30** **(0.13**–**0.73)**	**0.008**	0.74 (0.28–1.97)	0.55	1.36 (0.51–3.64)	0.54
Optimal control group therapy (vs. suboptimal)	0.19 (1.47–1.47)	0.23	0.58 (0.21–1.59)	0.29	1.69 (0.52–5.49)	0.38	0.73 (0.19–2.77)	0.64
Immunotherapy (vs. other)	0.71 (0.11–4.36)	0.71	1.37 (0.22–8.53)	0.73	2.26 (0.36–14.36)	0.36	0.87 (0.87–8.71)	0.91
Targeted therapy (vs. other)	0.99 (0.42–2.34)	0.98	0.66 (0.28–1.56)	0.34	0.54 (0.17–1.77)	0.31	0.37 (0.10–1.37)	0.14
Immunotherapy and targeted therapy (vs. other)	0.92 (0.41–2.08)	0.85	0.73 (0.32–1.65)	0.45	0.75 (0.27–2.11)	0.59	0.42 (0.13–1.35)	0.15
Chemotherapy (vs. other)	1.97 (0.95–4.80)	0.07	0.94 (0.46–1.95)	0.88	1.37 (0.56–3.38)	0.49	0.40 (0.16–1.02)	0.06
Hormone therapy (vs. other)	0.44 (0.13–1.50)	0.19	1.33 (0.42–4.24)	0.63	0.80 (0.20–3.29)	0.76	–	–

Statistically significant values are in bold.

*ESMO-MCBS* ESMO-Magnitude of Clinical Benefit Scale, *ASCO-VF* ASCO-Value Framework, *NCCN* National Comprehensive Cancer Network, *ASCO-CRC* ASCO-Cancer Research Committee, *OR* odds ratio, *CI* confidence interval, *OS* overall survival, *QoL* quality of life.

^a^Based on univariable logistic regression. All *P* values are 2-sided.

### Association between characteristics of applications and clinical trials and clinical benefit

Table 4 shows associations between characteristics of applications and of clinical trials with magnitude of clinical benefit. As expected, based on prior work^17,19^, there were statistically significant associations between high ESMO-MCBS scores and immunotherapy trials, drugs approved with a companion diagnostic test, breakthrough therapy designation, open-label trials, and studies which allowed crossover. However, for the ASCO-VF, only drugs with a companion diagnostic test and priority review were associated with greater clinical benefit. For NCCN Evidence Blocks, drugs with a companion diagnostic test were also associated with substantial clinical benefit while for ASCO-CRC statistically significant association with meaningful clinical benefit was observed with the use of intermediate endpoints.

**Table 4:** Association between characteristics of applications and clinical trial and clinical benefit.

	ESMO-MCBS		ASCO-VF		NCCN Evidence Blocks		ASCO-CRC	
	OR (95% CI)	*P* Value	OR (95% CI)	*P* Value	OR (95% CI)	*P* Value	OR (95% CI)	*P* Value
**Univariable analysis**^a^								
Immunotherapy (vs. other)	**2.61** **(1.36**–**4.99)**	**0.004**	0.69 (0.36–1.31)	0.26	1.36 (0.62–2.95)	0.44	0.89 (0.40–1.97)	0.76
Companion diagnostic (vs. not)	**2.97** **(1.52**–**5.78)**	**0.001**	**2.86** **(1.46**–**5.62)**	**0.002**	**3.53** **(1.37**–**9.11)**	**0.009**	1.50 (0.63–3.55)	0.36
Orphan drug (vs. not)	0.60 (0.33–1.12)	0.11	1.33 (0.72–2.46)	0.36	1.10 (0.54–2.26)	0.79	0.91 (0.42–1.96)	0.80
Breakthrough therapy (vs. not)	**2.69** **(1.35**–**5.36)**	**0.005**	**2.65** **(1.32**–**5.30)**	**0.006**	1.79 (0.79–4.07)	0.16	**2.75** **(1.03**–**7.29)**	**0.043**
Priority review (vs. not)	1.92 (0.85–4.37)	0.12	**3.98** **(1.61**–**9.83)**	**0.003**	2.18 (0.92–5.12)	0.08	**2.65** **(1.07**–**6.60)**	**0.036**
Palliative setting (vs. curative)	**0.04** **(0.01**–**0.29)**	**0.002**	**4.61** **(1.27**–**16.68)**	**0.020**	1.16 (0.34–3.98)	0.82	–	–
Initial approval (vs. supplemental)	**0.44** **(0.21**–**0.90)**	**0.024**	1.37 (0.69–2.70)	0.37	0.99 (0.44–2.22)	0.97	0.90 (0.39–2.06)	0.80
Regular approval (vs. accelerated)	7.95 (0.99–64.23)	0.05	2.72 (0.53–13.87)	0.23	3.68 (0.79–17.22)	0.10	0.54 (0.06–4.76)	0.58
One trial supporting approval (vs multiple trials)	0.56 (0.17–1.83)	0.33	0.48 (0.12–1.41)	0.16	0.49 (0.10–2.34)	0.37	0.59 (0.12–2.86)	0.51
Open-label (vs. double-blind)	**2.90** **(1.56**–**5.42)**	**0.001**	**1.96** **(1.06**–**3.61)**	**0.031**	1.46 (0.71–2.98)	0.30	0.86 (0.40–1.85)	0.70
Phase III (vs. phase II)	2.90 (0.77–10.94)	0.12	0.41 (0.12–1.41)	0.16	1.16 (0.34–3.98)	0.82	0.59 (0.12–2.86)	0.51
Sample size per 100 patients	1.05 (0.99–1.12)	0.12	**0.87** **(0.79**–**0.96)**	**0.004**	1.04 (0.95–1.15)	0.40	0.89 (0.79–1.01)	0.07
Approval based on subgroup analysis (vs. not)	0.92 (0.44–1.93)	0.83	1.70 (0.81–3.54)	0.16	1.21 (0.50–2.97)	0.67	1.12 (0.45–2.78)	0.81
Lung, breast, colorectal, and prostate cancer (vs. others)	1.58 (0.86–2.89)	0.14	1.20 (0.65–2.19)	0.56	1.47 (0.71–3.03)	0.30	1.49 (0.69–3.22)	0.31
Intermediate endpoint (vs. OS)	0.65 (0.35–1.20)	0.17	1.46 (0.78–2.74)	0.24	1.13 (0.55–2.34)	0.73	**2.42** **(1.11**–**5.28)**	**0.027**
Crossover (vs. not)	1.85 (0.91–3.75)	0.09	1.66 (0.82–3.35)	0.16	0.73 (0.32–1.67)	0.46	2.10 (0.78–5.66)	0.14
QoL benefit (vs. not)	–	–	–	–	4.50 (0.91–22.19)	0.07	2.20 (0.65–7.49)	0.21
**Multivariable analysis**^b^								
Immunotherapy (vs. Other)	**2.81** **(1.03**–**7.63)**	**0.043**	–	–	–	–	–	–
Companion diagnostic (vs. not)	**3.95** **(1.54**–**10.16)**	**0.004**	**3.82** **(1.67**–**8.78)**	**0.002**	**8.46** **(1.01**–**71.14)**	**0.049**	–	–
Breakthrough therapy (vs. not)	**2.63** **(1.08**–**6.41)**	**0.034**	1.87 (0.84–4.17)	0.13	–	–	1.76 (0.61–5.11)	0.30
Priority review (vs. not)	–	–	**3.64** **(1.14**–**11.68)**	**0.030**	2.77 (0.74–10.34)	0.13	2.36 (0.78–7.09)	0.13
Initial approval (vs. supplemental)	1.00 (0.39–2.59)	0.99	–	–	–	–	–	–
Regular approval (vs. accelerated)	**9.88** **(1.07**–**90.95)**	**0.043**	–	–	–	–	–	–
Open-label (vs. double-blind)	**2.85** **(1.22**–**6.66)**	**0.016**	1.45 (0.69–3.04)	0.33	–	–	–	–
Sample size per 100 patients	–	–	0.90 (0.81–1.00)	0.05	–	–	0.94 (0.81–1.08)	0.36
Intermediate endpoint (vs. OS)	–	–	–	–	–	–	**2.68** **(1.07**–**6.75)**	**0.036**
Crossover (vs. not)	**2.78** **(1.13**–**6.79)**	**0.025**	–	–	–	–	–	–
QoL benefit (vs. not)	–	–	–	–	2.32 (0.61–8.83)	0.22	–	–

Statistically significant values are in bold.

*ESMO-MCBS* ESMO-Magnitude of Clinical Benefit Scale, *ASCO-VF* ASCO-Value Framework, *NCCN* National Comprehensive Cancer Network, *ASCO-CRC* ASCO-Cancer Research Committee, *OR* odds ratio, *CI* confidence interval, *OS* overall survival, *QoL* quality of life.

^a^Based on univariable logistic regression. All P values are 2-sided.

^b^Multivariable models were adjusted for variables with *P* values < 0.10 in the univariable model.

## Discussion

RCTs have been the gold standard to demonstrate efficacy and safety of new cancer therapies. However, even RCTs have limitations^10–12^. The characteristics of control group therapy have been shown to influence the conclusions of RCTs^33^. Despite this, little is known about the influence of control group therapy on magnitude of clinical benefit scales. In this article, results show that among trials with substantial overlap between experimental and control therapy (e.g. active treatment plus a matched placebo in the control arm vs. the same active therapy plus an additional drug in the experimental arm) there appeared to be a lower odd of substantial clinical benefit with ESMO-MCBS and ASCO-VF, but no difference with NCCN Evidence Blocks and ASCO-CRC. We hypothesized that this discordance was explained by the difference in the methodology of these value frameworks. ESMO-MCBS and ASCO-VF grades are based on efficacy outcomes and adjusted if improvement in toxicity, QoL or tail of the curve effects are observed. It is important to highlight however that typically, these differences in QoL need to be statistically significant (despite generally low statistical power for such endpoints) and do not explore whether differences meet the minimally clinical important difference for the respective scales. NCCN Evidence Block scores are performed by NCCN Panel members and assess efficacy, safety, quality and quantity of evidence, consistency of evidence and affordability, whereas ASCO-CRC grades are applicable only in the non-curative setting and only evaluate efficacy (OS and PFS).

To investigate the influence of these methodologic differences in measurement of clinical benefit between scales, our post-hoc sensitivity analysis rescored trials with the ESMO-MCBS and ASCO-VF frameworks excluding data on QoL and high-grade toxicity. Results showed a non-significant and lower magnitude association between active treatment plus a matched placebo and lower clinical benefit. This suggests that QoL and toxicity likely explain at least part of the discordance observed between scales. These findings also have face validity as experimental therapy which is comprised in part by the same treatment as the control group would be expected to have a lower chance of reducing grade 3–4 toxicity and consequently QoL is less likely to be improved.

Consistent with prior studies^17–19^, our data show that less than a half of trials meet the threshold for meaningful clinical benefit as assessed using ESMO-MCBS and ASCO-VF, whereas approximately three quarters showed substantial clinical benefit using the NCCN Evidence Blocks and the ASCO-CRC criteria. Of note, the type of active therapy was not associated with statistically significant differences in the magnitude of clinical benefit. However, this analysis was limited by small sample sizes, and it is noteworthy that meaningful effect sizes were observed for control groups comprising of chemotherapy (ESMO-MCBS and NCCN Evidence Blocks) and of immunotherapy (NCCN Evidence Blocks).

The appropriateness of control group therapy was not associated with a statistically significant difference in the magnitude of clinical benefit. There seemed to be non-significant association with lower odds of substantial clinical benefit for trials with optimal control groups when using the ESMO-MCBS and ASCO-VF while a non-significant effect in the opposite direction was observed for NCCN Evidence Blocks. The observation that these associations were in opposite directions again suggest the importance of QoL and high-grade toxicity assessment in the interpretation of the results of these frameworks. Of interest, the ASCO-CRC framework was not sensitive to this effect, and this could be explained by the fact that this framework can only be applied in the palliative setting and its assessment is based exclusively on efficacy outcomes.

Of interest, we also explored predictive factors associated with clinical benefit. Consistent with prior studies^17^, trials supporting approval of cancer drugs with a companion diagnostic test were more likely to be scored as having a substantial clinical benefit according to the ESMO-MCBS, the ASCO-VF and the NCCN Evidence Blocks. This observation which has been reported previously^17,19^ is likely explained by higher magnitude of benefit seen when targeted therapy is delivered to groups of patients most likely to benefit for it and avoiding empirical exposure (and thereby unnecessary toxicity) of those who are unlikely to benefit^34^. In addition, some variables such as immunotherapy trials, breakthrough therapy designation and priority review were associated with substantial clinical benefit. Of note, consistent with prior work^17,19^, our analysis showed an association between higher framework scores and intermediate endpoints. Regulators require clinical trials to show that surrogate endpoints can be relied upon to predict, or correlate with, clinical benefit^35^. As not all endpoints which were examined in our analysis met the above definition, we elected to use a broader term of intermediate endpoint which we believe is the more scientifically robust term. The observation that potentially unvalidated endpoints are associated with higher clinical value scores is an area of concern. Furthermore, the discordant observation of palliative setting and clinical benefit with ESMO-MCBS and ASCO-VF is likely explained by the different ways in which trials are assessed by these frameworks. In the palliative setting (which comprises the majority of included trials), studies may have higher odds of substantial clinical benefit with ASCO-VF due the ability to apply extra points cumulatively for outcomes such as tail of the curve effects, treatment free interval, cancer-related symptoms and QoL. In contrast, with ESMO-MCSB a total of 1 extra point can be added to such effects.

Our study has several limitations. First, we evaluated clinical benefit at the time of approval, however, the analysis of clinical benefit can change over time with updated data on efficacy, toxicity or QoL over the course of post-marketing period^17,36^. Second, the source for data collection was variable with QoL and patient-reported outcomes data being extracted from published articles rather than drug labels. Unfortunately, these data are frequently not presented in primary publications of clinical trials. Third, defining control therapy group as optimal and suboptimal might be controversial given that depending on the tumor type, optimal treatment can rapidly evolving. Fourth, our assessment of whether control group therapy was optimal was based on standards of care in high income countries. Value frameworks are utilized often in lower resource environments where these definitions of optimal control group therapy may not apply. Finally, in many of the analyses, the sample size was small. This resulted in an inability to adequately fit multivariable models. This will add some uncertainty to the reported results and thereby limit generalizability.

In summary, clinical benefit scales can be sensitive to the type of control group therapy. RCTs with an active treatment plus matched placebo in the control arm were less likely to be scored as providing substantial clinical benefit using the ESMO-MCBS and the ASCO-VF scales. Control group therapy did not influence NCCN Evidence Blocks or ASCO-CRC scores. This is likely explained, at least in part, by differences between the different clinical benefit scales in the inclusion and/or weighting of QoL and toxicity. These results can be used to aid in the design of clinical trials (trials with substantial overlap between experimental and control therapy are likely to have a lower effect size and attenuated impact on QoL assessment), inform drug reimbursement decisions by payers (a lower incremental cost effectiveness ratio would likely be observed with greater overlap between experimental and control group) and provide feedback to the developers of value frameworks. The sensitivity of the ESMO-MCBS and ASCO-VF frameworks to control group therapy should be taken into consideration for future development of these scales. Adjustment of scores of trials with overlapping treatments may be warranted.

## Data Availability

The data that support the findings of this study are available upon request from the corresponding author (EA).
